# Prophylaxis: Extension *for* Prevention, or Extension *of* Prevention?

**Published:** 1903-11

**Authors:** Lyman Curtis Bryan

**Affiliations:** Basel, Switzerland


### PROPHYLAXIS: EXTENSION *FOR* PREVENTION, OR EXTENSION *OF* PREVENTION?[1]

BY LYMAN CURTIS BRYAN, D.D.S., F.B.D.C., BASEL, SWITZERLAND.

THE undoubted increase from generation to generation of the ravages of decay in the human teeth leads us to pause in our methodical ways of treating and filling teeth, even though we have made the most approved methods of modern dentistry our own, and ask ourselves if we are doing the best of which we are capable for our patients and getting the best possible results.

It will be conceded that it is the usual thing for the dentist and patients to meet each other annually or semiannually and to find, almost to a mathematical certainty, that there are a number of cavities that require filling. In a practice where patients are well

---

[1] Read before The New York Institute of Stomatology, May 5, 1903.

trained—among intelligent people—they have been led to expect this and to think that it is unavoidable. The best patients do not wait until they experience pain—they come regularly for examination, and expect the dentist will find cavities and fill them before the pain occurs. They know that cavities left until then are more painful to fill, require longer time to treat, and cost them much more to repair, with greater danger of giving trouble afterwards.

They find it economy in time, money, and pain to go to the dentist often, as often as he thinks fit, and will come exactly at the time recommended. A patient who does so, and who brushes his teeth from one to three times a day, has a perfectly clear conscience as regards his teeth, and thinks he is doing his whole duty. If the dentist fills the cavities present and lectures the patient on more cleanliness or the use of silk between the teeth, proper brushes, and an antiseptic wash, and then shines up the front teeth with a brush-wheel and removes the calculus from the sixth-year molars above and the six incisors below, he too has a clear conscience as to that particular patient. He finds even these first-class patients begin to have recession of the gums later in life, slight discharges of pus may occur about the necks of certain teeth, the gums may become slightly festooned or swollen, or the teeth may get loose in their sockets.

Then he tells the patient that he has pyorrhœa alveolaris, probably inherited from his parents, or it may be the result of overwork or overworry, or, lastly, of sanitary conditions. We do not think for a moment that it has been our fault, neither does the patient think it is his fault; we all take it as an indication of increasing years and the result of our environment or of predisposing causes. We could have prevented it. We see cavities, some below or about perfect gold or amalgam fillings; we fill these and advise stronger brushing and other prophylactic treatment by the patient, which he probably thinks is superfluous advice, as he brushes his teeth three times a day and does not think he can possibly devote more time to it.

The next time he comes we have an approximal cavity, say, on the distal surface of a molar, to fill for this patient. We then enlarge the cavity to cover a space which extends to the line reached by the brush, by cutting away with a chisel all frail walls and extending our cavity as Professor Black has (and as Professors Wetherbee and Coolidge) advised, in my college days, beyond the point of danger. This is "extension for prevention."

This does protect that particular surface of that particular tooth from further decay, but there are at least five other surfaces subject to decay on that molar. The patient may think we are cutting away from a good tooth to make a large cavity, especially if we use some force with our chisel and break off pieces of enamel which appear to him to be strong. Some ignorant people imagine the dentist makes cavities to fill them, or makes those they have larger to get a larger fee; but we know we have done our duty; we know "Extension for Prevention" is the watchword of the day and is approved by the best men in the profession; so we feel that we are doing the best we can for the patients. We have made a large contour filling that we know will remain a monument to our skill for many years, and we stretch ourselves up in pride and pat ourselves on our lame back and say to ourselves, "Well done, dear old fellow!" Dr. Blank never made as fine a filling as that, and we think we have done the best we could for our patient. But lo! next year there is a cavity on the mesial surface of that tooth and three or four other cavities in the mouth requiring the same laborious, painstaking, and painsmaking care to extend and prevent future trouble in each particular case, and so it goes on from year to year, and the dentist's work is never done.

We have come to consider this the working of natural laws. We have probably thought that we should devote more time to prophylaxis, more scraping and polishing, removing of slightly affected spots on the teeth, flooding them afterwards with a forty per cent. solution of nitrate of silver and polishing away the discoloration again, painting discolored surfaces of teeth with iodine and polishing them bright with pumice and wheels, soaking nitrate solution in around margins of fillings where decay is liable to occur and between teeth which lie closely together and present rough surfaces where the silk passes through, trimming up margins of fillings which irritate the gums and hold decay-producing débris; but after we have had the patient several sittings to make our great operations and do what he recognizes must be done, he is glad to get out of our hands, and we have not the heart to say to him, "Much of this work which we have lately done could have been avoided by doing a number of little things for prevention. It is possible for you and me to keep your teeth in such a state of cleanliness and antisepsis that much of this decay would not occur. If I could now spend an hour or two more for you in doing little things which we class under the heading of prophylaxis, your mouth would be in a much better condition to resist decay and next year we would not have so much to do."

No; we see he is tired of it all and glad to get away. We may have scraped tartar off in a hurry and wounded his gums and made the necks of his teeth so sensitive that he will remember us for a week with regret; we know his bill will be large anyway, and perhaps he would pay less willingly the five or ten dollars under the heading "various operations," or "cleansing the teeth," after all the other items, which he finds large enough as it is, and he will not understand why he should pay us as much per hour for "fussing around and killing time" as for making a gold filling, which he knows he had to have made to save the tooth and prevent toothache.

But a trumpet voice sounded in Philadelphia that is reverberating through America in unmistakable tones and echoing around the world, making for many of us an uncomfortable spot in our moral anatomy where our conscience is supposed to lie, Dr. Smith and his apostles tell us we are not doing our duty by our patients. This band of reformers of dental practice tell us we have no right to let people go on in ignorance of prophylactic treatment, or of what can be done to prevent decay; of what their duty is to their teeth and what our duty is to them. We are told we can prevent decay in many places. We should do it. We should see our patients oftener and make fewer large operations; do them also when necessary, but in time bring the tooth up to a higher plane of vitality; attend to the little things like those mentioned above which experience has taught us will prevent decay in certain places. We should spend twice the time and care in treating and cleansing the teeth.

We should use nitrate after drying the teeth, so that it will penetrate and stimulate the tooth to resist decay. We should use iodine to discolor the injurious films and loosen them up about the teeth where decay comes; this we should polish off with pumice powder until the teeth are clean on every surface above and below the gums that we can reach with hand wood-points, engine-points, thin tape, and silk thread covered with powder between the teeth. Places already softened and decalcified should not be pointed out to the patient with the remark, "Next year you will have a cavity there," or "Come in six months to have this place filled." We should remove the decalcified surface, dry it, paint it with nitrate of silver, and afterwards remove the discoloration with iodine tincture (producing probably ioduret of silver, which is yellowish white).

We should cleanse with delicate scalers—Younger's and other fine, smooth-pointed ones—the neck and under the gum of each tooth, hunting for those little commencements of pockets about the necks and use in them our caustics and stimulants, and not simply tell a patient that in five years he will have pus-discharging pockets and loose teeth. Our first duty should be to thoroughly cleanse the teeth before starting to fill or do other work.

As demonstrator in charge at the Boston Dental College I never allowed a student to commence filling teeth for a patient until he had thoroughly cleansed the teeth. This they considered a great hardship, and shirked it as much as they could; and, judging from most new patients I see, the general run of dentists avoid this as much as my students used to.

Many dentists who spend a half-hour doing this, at most an hour, will assure you that they do this most thoroughly, but a specialist in pyorrhœa will spend hours before he has got a mouth with calculus on the roots clean.

We should gradually induce our regular patients to come often for extension of prevention, and we shall have less extension for prevention. We shall have to practise both, but the time will come when one can point with pride to the glistening, knuckling molars and bicuspids, with their surfaces as nature made them, as we point now to the glistening and golden monuments of our misguided efforts and neglected duty to our patients.

This is an ideal future, and will not be for us, who have done our half- or quarter-century of work, to see its full fruition. Its perfect results will only be seen by the younger men of the profession. It will have to be done by commencing with the young—the very young, and we cannot expect to see results save after a few years, but I feel a solemn faith in the future of extension of prevention, or prophylaxis, in its various branches. If we use all the known methods ourselves, and use them often, seeing patients much oftener than we have done and some as often as Dr. Smith recommends for his hand, wood, and pumice treatment, "once a month," and others every three months when the teeth are weak and decay often occurs and recurs, we shall more certainly be doing our duty by our patients, and there will come a time—which Dr. Smith tells us he has already reached—when decay will be the exception and the teeth will be strengthened and protected, not simply repaired and patched.

The oftener we see our patients and interest them in their teeth the more personal care they will take of them. We are told that in a short time teeth so treated will take on an entirely different nature of enamel and dentine; that the tooth is stimulated by rubbing with wood and pumice powder just as the muscles are by massage; that the process of building and repair which goes on in the tooth from the pulp will be stimulated inside, to resist decay from outside. We know that nitrate of silver not only kills the germs on the surface and makes the tooth immune for a time, but it has the stimulating power of an irritant, and causes the pulp in the tooth to throw out a protecting wall of dentine of very dense structure which resists decay. We further know that nature carries out this same process to a certain extent in every cavity of decay, but under present conditions decay overcomes this process of repair and goes on in spite of it. We know that the tooth is almost certain not to decay when it is kept perfectly and constantly clean or treated with nitrate of silver. We know that teeth only decay on the surfaces which it seems impossible to keep clean and which are not kept clean. We know that the teeth which look clean have invisible coatings of deleterious and injurious germ cultures, and now we know that it is our duty to remove these often and thoroughly. Do we do it? We should do it. If we cannot do it as often as it should be done, we should use the nitrate of silver solution everywhere every time we see the patient. No fear need be felt of discoloring even front teeth. Enamel which is not disintegrated will not take on the dark color from it. If it does, then we have a danger signal there; the coming trouble is located; we can remove the discoloration with iodine, and any traces of iodine or its combination with nitrate; with ammonia; with iodine painted on the silver stain, and ammonia to wash or scrub off the iodine. Nitrate stains, when fresh, can be removed from the fingers also, and dark remnants are rubbed off with a block of pumice-stone or sapolio. Rubber gloves used by surgeons may be used to protect the hands. I have families of children with frail teeth, which I bathe, from incisors to molars, in nitrate of silver forty per cent. as often as I can see them, and I am having splendid results with them and no serious discoloration. It is only latterly that I have extended this treatment to the incisors. I have been using it in increasing quantities and more frequently for several years since Dr. Stebbins recommended it so fully.

I had a family in which the mother's teeth had just melted away, and she and her sisters had almost all the teeth back of the cuspids crowned or bridged. As the younger generation came on, I began the use of nitrate on the temporary and permanent molars, and with a little girl, who is now ten years old, I had succeeded so well in saving these molars and other teeth that I was congratulating myself that I had stopped decay in them. Recently I happened to think it was time to begin to clean the lower incisors, which were all erupted. What was my horror to find them all decayed,—six cavities, large ones, in the four new little incisors! Such a case of early decay I had never seen before. I had noticed signs of decay in the upper permanent incisors, and had bathed them freely in nitrate and had kept them from decay approximately, but had never seen the lower incisors decayed at that age. One can think of the beneficial effects of nitrate, by its saving all the other teeth, while these little unsuspected teeth, that almost never decay until all others are filled or crowned, had become damaged under my very eyes.

I have often thought, since Dr. Rosenthal, of Brussels, suggested the abonnement system (Dr. Smith had used it in certain cases), that we should have a system of abonnement for children, for in this way we could get our little patients to come often, and we could demand their frequent visits, and their parents would not think we were doing unnecessary work or seeing them unnecessarily often. As it is, with our fee system, many of us would hesitate to demand the attendance of our patients who have frail teeth as often as it is necessary, for fear they would think we had little to do and wished to make capital out of them. I believe it would be a good system to propose to a certain class of regular patients to take a general average of their work for the last years, agree to treat their teeth for less or for an equal annual sum, and then have them come as often as we feel we could benefit them and as often as was necessary to thoroughly apply our prophylactic treatment.

The first year our time would not be so fully paid, but from the second year the results would be a decided gain for both parties, for we could prevent decay in less time than we could repair and remedy it, and the patient would be saved much pain and have infinitely better teeth and general health, besides less display of gold and other fillings. Unquestionably pyorrhœa would be less frequent and troublesome, and we should see the advantages of extension of prevention over extension for prevention.

Dental friends to whom I have proposed this treatment object that the great advantages of this preventive treatment would not be appreciated by patients and they would not pay for it as readily as for filling cavities which came in teeth as has been our custom; that it is not our duty to prevent cavities and reduce our work and income; but there are others who look at the subject from a higher plane, and will be more conscientious in treating patients who confide their teeth to us and expect us to do our best to save these, to save them pain, to strengthen weak teeth, to prevent the loss of teeth in later life through Rigg's disease, or pyorrhœa, to prevent the loss of pulps and resulting abscesses, etc.

We are told and believe that prophylaxis secures almost immunity from decay, the great improvement in color and general appearance of the teeth, the diminished sensitiveness of the dentine, the tightening of many teeth which had become loose, the relief from undue sensitiveness of the gums, their marked adhesion to the necks of the teeth, the beautiful color and striation appearing in them, the cleanliness and general comfort of the mouth, and the universal improvement in the character of the breath. These are all matters attracting notice, inspiring confidence, and awakening most lively interest.

Dr. Smith, in his able articles on his special treatment by hand with wood-points and coarse pumice-stone powder applied once a month in certain cases, says, "Recognition will yet be made of the important fact that to the presence of foreign matter on and about the teeth, rather than to the quantity of it, the beginnings of decay and pyorrhœa are wholly attributable. The deleterious influence of a breath perpetually laden with offensive emanations from this source, especially during seasons of salivary inactivity, as in sleep, will ere long be disclosed as an important factor in many pulmonary and digestive disorders, and will be taken account of in medical diagnosis and treatment."

